# Maxillofacial trauma among Indians

**DOI:** 10.6026/97320630019876

**Published:** 2023-08-31

**Authors:** Saurabh Kumar, Sandeep Kashyap, Saurabh Singh, Rohit Sharma, Yatendra Pratap Singh, Hani Yousuf Naik

**Affiliations:** Department of Dentistry, Public Health Center, Bind, Nalanda, Bihar Government, Bihar, India; Department of Dentistry, Sikkim Manipal University, Gangtok, Sikkim, India; Department of General Surgery, Rama Medical College Hospital And Research Centre, Kanpur, Uttar Pradesh, India; Department of Oral and Maxillofacial Surgery, School of Dental Sciences, Sharda University, Greater Noida, Uttar Pradesh, India; Department of Otorhinolaryngology, Saraswati Medical College, Unnao, Uttar Pradesh, India; Department of Oral and Maxillofacial Surgery, Pacific Dental College and Research Centre, Udaipur, Rajasthan, India

**Keywords:** Assault, maxillofacial fractures, maxillofacial trauma, orofacial trauma, retrospective analysis, road-traffic accident

## Abstract

Orofacial injuries constitute the medico-legal cases reported, especially, in cases associated with road traffic accidents, assaults, and violence making it an emerging healthcare problem. Therefore, it is of interest to document data on the maxillofacial trauma and fractures among Indians. 150 subjects within the age of 15 to 60 years with maxillofacial fractures, detailed medical history including demographics, radiographs, medical history, associated injuries, and etiology of fractures were used for this study. Sites for both maxillary and mandibular fractures were noted. The type of intubation (medical insertion procedure) used and post-operative complications were also recorded. Lefort I, II, and III fractures were seen in 4%, 12%, 6% subjects respectively, whereas, ZMC fracture was seen in 66% study subjects. Mandibular fractures were most commonly seen in the para-symphysis region with 30% subjects followed by condylar region with 28.66% subjects. Data shows that maxillofacial trauma has a high incidence in India with RTA (road traffic accidents being the most common reason for the trauma seen in young males with significant concomitant injuries. Most common fracture is seen in mandible region. However, they can be managed well with very few postoperative complications.

## Background:

Maxillofacial trauma is increasing in incidence and is commonly seen in assaults, emergency, and accident cases reported to hospitals [1]. Orofacial injuries with increasing incidence globally constitute the medicolegal cases reported, especially, in cases associated with road traffic accidents, assaults, and violence making it an emerging healthcare problem [2]. Maxillofacial trauma and injuries have varied etiologies depending on the geographic area assessed, localities within the same geographic areas which are largely governed by the environmental, cultural, and socioeconomic status of the individuals with orofacial trauma [3]. Proper and critical assessment of maxillofacial fractures and trauma in India can help in the assessment of trauma patterns and can provide insight into finding appropriate preventive measures to reduce the incidence of maxillofacial trauma and injuries [4]. Various previous literature data has focused on the Etiology and severity of maxillofacial injuries with the varied incidence in different geographic regions of India. These studies depict the association of maxillofacial injuries mostly to assault and road traffic accidents. However, the concerning data is scarce and unclear [5]. Therefore, it is of interest to assess the pattern, etiology, intubation mode, associated complications, and outcomes following the management of maxillofacial trauma in tertiary care hospital in India. It is also of interest to obtain a clear picture of demographics, epidemiology, and etiology of maxillofacial injuries.

## Materials and Methods:

## Study design:

The retrospective clinical study was aimed to assess the pattern, etiology, intubation mode, associated complications, and outcomes following the management of maxillofacial trauma in subjects reporting to a tertiary care hospital in India. The study also aimed to obtain a clear picture of demographics, epidemiology, and etiology of maxillofacial injuries. The study was preceded after the ethical clearance from Rama Medical College and Hospital Kanpur. The study subjects were recruited from the patients admitted to Department of Emergency and Trauma, Rama Medical College and Hospital, Kanpur and were diagnosed with maxillofacial fracture post-surgery. The study assessed 150 subjects, both males and females in the age of 15-60 years. The mean age of the participants was 32.43±6.28 years.

## Methodology:

**Figure 1:**
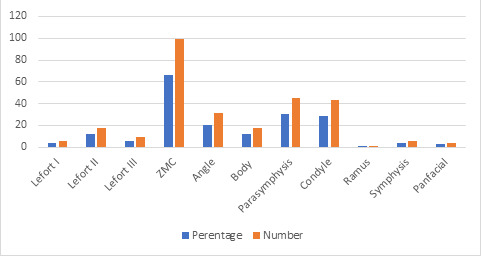
Sites distribution of the maxillofacial trauma

**Figure 2:**
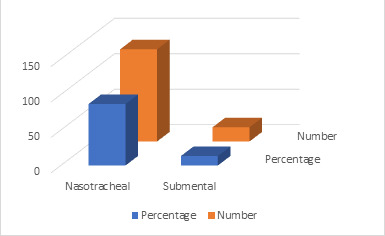
Type of Intubation in study

The inclusion criteria for the study were subjects with a confirmatory diagnosis of maxillofacial both clinically (Figure 1) as well as radio-graphically (Figure 2), history of trauma, subjects treated for fracture at the institution, and subjects willing to participate. Following inclusion of the participants, medical records of the subjects were extracted from the institution records. Before treating subjects for the maxillofacial fractures, inter-disciplinary coordination was done with general surgeons, neurosurgeons, ENT, and emergency trauma care team in subjects with the associated injuries with the maxillofacial trauma. To prevent bias, the records were checked by the two different investigators, and any associated disagreement was discussed and agreed upon. The data collected were detailed medical history including demographics, radiographs, medical history, associated injuries, and etiology of fractures. Fracture sites for both maxillary and mandibular fractures were noted. The type of intubation used and postoperative assessment was also recorded. Any reported post-operative complication was also taken into account.

## Data analysis

The data collected were analyzed statistically to formulate the results. The data were expressed as percentage and number, and mean and standard deviation.

## Results:

As shown in Table 1, majority of the study subjects were within the age range of 26-35 and 36-45 years with 32% (n=48) and 34% (n=51) subjects respectively. There were 67.33% (n=101) males and 32.66% (n=49) females in the study. The causes for the fracture were road traffic accidents, fall from height, assault, sport injuries (cattle dash), and alcohol influence in 66.66% (n=100), 14.66% (n=22), 8.66% (n=13), 6.66% (n=10), and 3.33% (n=5) subjects respectively. Concomitant injuries seen were head injuries, pelvis, chest, spine, orthopedic injuries, and abdomen injuries in 64% (n=96), 3.33% (n=5), 10% (n=15), 1.33% (n=2), 15.33% (n=23), and 6% (n=9) subjects respectively.

It was noted that in maxillary and mid face fractures, Lefort I, Lefort II, and Lefort III fractures were seen in 4% (n=6), 12% (n=18), 6% (n=9) subjects respectively, whereas, ZMC fracture was seen in 66% (n=99) study subjects. Mandibular fractures were most commonly seen in the para-symphysis region with 30% (n=45) subjects followed by the condylar region with 28.66% (n=43) subjects. Angle fracture was seen in 20.66% (n=31) subjects followed by body fracture in 12% (n=18) subjects, symphysis fracture in 4% (n=6) subjects, and ramus fracture in 0.66% (n=1) study subject. Pan facial fracture was seen in 2.66% (n=4) of study subjects (Table 2 and Figure 1).

**Figure 3:**
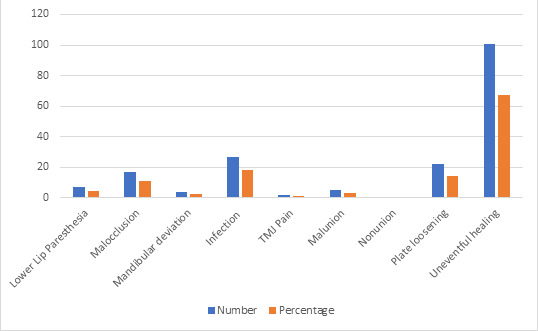
Postoperative complications in the study

Data shows that nasotracheal intubation was done in 86.66% (n=130) study subjects, whereas, sub mental intubation was done in 13.33% (n=20) study subjects (Table 3 and Figure 2). Sub mental intubation was done in all subjects with Panfacial fractures. The postoperative complications were also assessed in the study subjects. Uneventful and complete healing was seen in 67.33% (n=101) of study subjects. Most common complication seen was plate loosening seen in 14.33% (n=22) study subjects followed by infection in 18% (n=27) subjects, malocclusion in 11.33% (n=17) subjects, transient paresthesia of lower lip in 4.66% (n=7) subjects, mal-union in 3.33% (n=5) subjects, mandibular deviation in 2.66% (n=4) subjects, and pain in TMJ in 1.33% (n=2) study subjects. Non-union was not seen in any study subject (Table 4 and Figure 3).

##  Discussion:

The present clinical retrospective study was conducted to assess the pattern, etiology, intubation mode, associated complications, and outcomes following the management of maxillofacial trauma in subjects reporting to a tertiary care hospital in India. The etiology of maxillofacial injuries is known to vary from one geographical region to another. In developing countries, road traffic accident is generally believed to be the most common cause of facial trauma [6] and this has been confirmed by some of the previous studies [7 8,9]. Trauma is associated with significant morbidity and mortality in individuals. Maxillofacial (MF) injuries may lead to functional impairment and aesthetically altered appearance if not attended promptly. Factors like the geographic area, population density socioeconomic status, and the cultural variances amongst the study population have influenced the incidence etiology and pattern of maxillofacial injuries since ages [10,11, 12,13,14]. The study results showed that in maxillary and midface fractures, Lefort I, Lefort II, and Lefort III fractures were seen in 4% (n=6), 12% (n=18), 6% (n=9) subjects respectively, whereas, ZMC fracture was seen in 66% (n=99) study subjects. Mandibular fractures were most commonly seen in the parasymphysis region with 30% (n=45) subjects followed by the condylar region with 28.66% (n=43) subjects. Angle fracture was seen in 20.66% (n=31) subjects followed by body fracture in 12% (n=18) subjects, symphysis fracture in 4% (n=6) subjects, and ramus fracture in 0.66% (n=1) study subject. Pan facial fracture was seen in 2.66% (n=4) of study subjects. These results were comparable to the results by Malara *et al.* [15] In 2006 and Jarius [16] in 2008 where authors compared similar demographics, reported similar etiology, and associated injuries as in the present study. Concerning the intubation performed, nasotracheal intubation was done in 86.66% (n=130) study subjects, whereas, sub mental intubation was done in 13.33% (n=20) study subjects. Sub-mental intubation was done in all subjects with Panfacial fractures. This was in agreement with the studies of Hall C *et al.* [17] in 2003 and Vasishta *et al.* [18] in 2010 where the comparable incidence of using naso-tracheal and sub-mental intubation was reported. In the present study, postoperative complications were also assessed. Uneventful and complete healing was seen in 67.33% (n=101) of study subjects. Most common complication seen was plate loosening seen in 14.33% (n=22) study subjects followed by infection in 18% (n=27) subjects, malocclusion in 11.33% (n=17) subjects, transient paresthesia of lower lip in 4.66% (n=7) subjects, mal-union in 3.33% (n=5) subjects, mandibular deviation in 2.66% (n=4) subjects, and pain in TMJ in 1.33% (n=2) study subjects. Non-union was not seen in any study subject. These complications were similar to what is reported by Zweig [19] in 2009 and Pham-Dang *et al.* [20] in 2014 where similar post-operative complications as of the present study were reported by the authors.

## Conclusion:

Data shows that maxillofacial trauma has a high incidence in India with road traffic accidents being the most common reason for trauma seen among young males with significant concomitant injuries. Most common fracture seen is in mandible region. However, they can be managed well with very few postoperative complications. Ensuring proper traffic rules following and setting dedicated maxillofacial trauma centres can help in reducing the incidence and ensure effective management.

## Figures and Tables

**Table 1:** Demographic characteristics of the study subjects

**S. No**	**Characteristics**	**Percentage (%)**	**Number (n)**
1.	Mean Age	32.43±6.28	
2.	Age Range	15-60	
a)	15-25	22	33
b)	26-35	32	48
c)	36-45	34	51
d)	46-55	7.33	11
e)	>55	4.66	7
3.	Gender		
a)	Males	67.33	101
b)	Females	32.66	49
4.	Fracture Cause		
a)	Road Traffic Accidents	66.66	100
b)	Fall from Height	14.66	22
c)	Assault	8.66	13
d)	Sports Injuries (Cattle dash)	6.66	10
e)	Alcohol Influence	3.33	5
5.	Concomitant Injuries		
a)	Head Injuries	64	96
b)	Pelvis	3.33	5
c)	Chest	10	15
d)	Spine	1.33	2
e)	Orthopedic	15.33	23
f)	Abdomen	6	9

**Table 2:** Sites associated with the maxillofacial trauma in study subjects

**S. No**	**Fractures**	**Percentage**	**Number (n=150)**
1	Maxillary and midfacial Fractures		
a	Le Fort I	4	6
b	Le Fort II	12	18
c	Le Fort III	6	9
d	ZMC fracture	66	99
2	Mandibular Fractures		
a	Angle	20.66	31
b	Body	12	18
c	Parasymphysis	30	45
d	Condyle	28.66	43
e	Ramus	0.66	1
f	Symphysis	4	6
3	Pan facial fractures	2.66	4

**Table 3:** Type of Intubation performed in subjects with maxillofacial trauma

**S. No**	**Intubation**	**Percentage**	**Number (n=150)**
1	Nasoendotracheal intubation	86.66	130
2	Submental Intubation	13.33	20

**Table 4:** Complications of maxillofacial fracture treatment in the study subjects

**S. No**	**Post-operative complications**	**Percentage (%)**	**Number (n)**
1.	Transient Paresthesia (Lower lip)	4.66	7
2.	Malocclusion	11.33	17
3.	Mandibular deviation/deflection	2.66	4
4.	Infection	18	27
5.	Pain in TMJ	1.33	2
6.	Malunion	3.33	5
7.	Non-union	0	0
8.	Plate Loosening	14.66	22
9.	Uneventful healing	67.33	101
